# Transvesical removal of huge seminal vesicle cystadenoma

**DOI:** 10.11604/pamj.2017.28.149.14003

**Published:** 2017-10-17

**Authors:** Mojtaba Ameli, Naser Yousefzadeh

**Affiliations:** 1 Gonabad University of Medical Sciences, Gonabad, Iran; 2 Iran University of Medical Sciences, Tehran, Iran

**Keywords:** Cystadenoma, transvesical, seminal vesicle

## Abstract

Benign tumors of the Seminal vesicle that occur more commonly then malignant tumors. Cystadenoma is an extremely rare benign tumor of the seminal vesicle. Management of these tumors remains debatable due to the limited data in the literature. We present a cystadenoma of the seminal vesicle was resected using an open transvesical approach. A Sonography reported enlarged prostate with volume of 560 ml with multiple cystic area inside it. CT scan and abdomen and pelvic MRI with and without Gadolinium also performed and huge (120 * 100 * 100mm) heterogeneous mass in anatomic site of prostate reported. Patient underwent pelvic mass resection by retro vesical abdominal approach. Final pathology report is consistent with cystadenoma/epithelial stromal tumor of the seminal vesicle with no suggestion for malignancy. After recovery from surgery patient was symptom free and totally continent with preserved erectile function. Surgical intervention may be considered when a cystadenoma of the seminal vesicle is diagnosed and symptoms or tumor growth occurs transvesical removal of seminal vesicle mass is an effective surgical procedure for seminal vesicle disease.

## Introduction

The seminal vesicles are paired viscous organs that are positioned posterior to the bladder and prostate. It has capacity for 3-4 CC and non-obstructive seminal vesicle typically measures 5-7cm in length and 1.5cm in width. Seminal vesicles secrete 80% of seminal fluid [1]. Because of the anatomic location, surgical access and management of seminal vesicle pathology can be difficult for the urologist. High-resolution trans rectal ultrasonography (TRUS) has become the mainstay of imaging for the diagnostic evaluation of seminal vesicle pathology. Hyper-echoic solid masses in the seminal vesicles revealed on TRUS are concerning for tumor. If there is a unilateral solid mass, it is more likely to be a primary tumor and TRUS guided biopsy and aspiration is necessary to assist in the diagnosis. CT scan and MRI could be useful to characterize the lesion of Seminal vesicle. Benign tumors of the seminal vesicle that occur more commonly then malignant tumors, include, fibromas, leiomyosarcomas, cystadenomas, schewannomas and papillary adenomas [1]. Here is a case of cystadenomas of seminal vesicle, which is detected on final pathology of middle-aged, came to our center with lower urinary symptoms and a huge pelvic mass.

## Patient and observation

**Figure 1:**
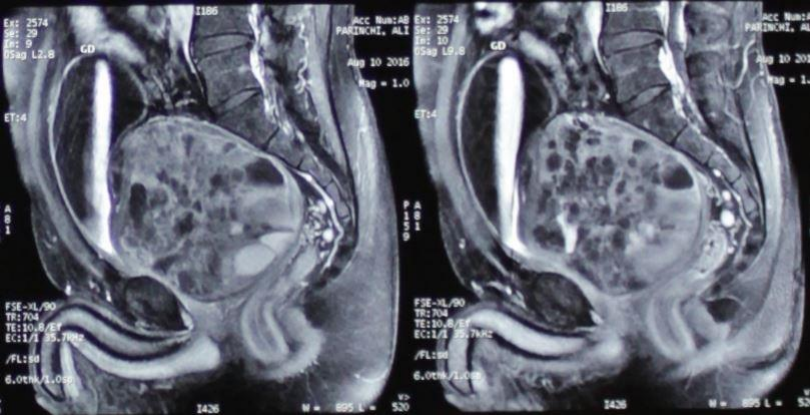
MRI of abdominopelvic

**Figure 2:**
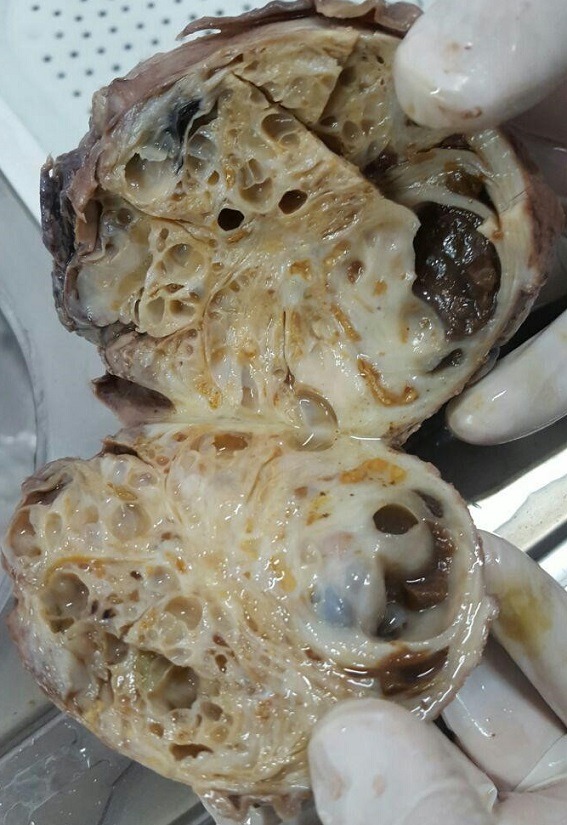
An overview of the mass

A 49 years old man, came as outpatient to the clinic with complain of frequency since 5 months ago. He also mentioned dysuria, mild obstructive urinary symptoms and constipation, with gradual onset, initiating since 6 months ago. He didn't have other urinary symptoms, hematuria, flank pain or weight loss. Past medical and surgical history was negative. On physical examination, all organ systems were normal except for huge prostate, palpable at digital rectal exam which was rubbery and symmetric. We had started our work up by checking renal function test, PSA level and sonography of kidneys and prostate. All laboratory data came back within normal ranges except for PSA which was 11.2. Sonography reported enlarged prostate with volume of 560ml with multiple cystic area inside it. Post void residue was zero. CT scan and abdomen and pelvic MRI with and without gadolinium also performed and huge (120 * 100 * 100mm) heterogeneous mass in anatomic site of prostate reported (Figure 1). The mass seen with solid and cystic component, well circumscribed and contained partially thick peripheral capsular. There was no adjacent structure involvement. Patient underwent pelvic mass resection by retro vesical abdominal approach. The mass which has elongation beside prostatic urethra revealed. By help of frozen section we got ensured that it has benign nature, 70% of mass freed from adjacent structures. Due to its extension near prostatic urethra and risk of external sphincter injury or neurovascular bundle injury, we spared part of its capsule (which was elongated near prostatic urethra) but enucleated inside it (Figure 2). After recovery from surgery patient was symptom free and totally continent with preserved erectile function. Final pathology report is consistent with cystadenoma/epithelial stromal tumor of the seminal vesicle with no suggestion for malignancy.

## Discussion

Primary diseases of the SVs are very rare. Benign tumors can appear as complex, solid cystic retro vesical masses and are often totally asymptomatic. However, they can also lead to LUTS and unspecific signs, such as hematuria, hematospermia, perineal or postcoital discomfort and painful defecation. Occasionally, infertility will be the main feature [2]. Cystadenoma is an extremely rare benign tumor of the seminal vesicle with only 15 cases reported so far in the English literature [3]. Cystadenomas of the Seminal vesicle typically occurs in middle-aged man and are almost never bilateral and diagnosis is typically made on final pathology after surgical resection. Surgical approach to the Seminal vesicles depends mainly on the expertise and comfort of the surgeon, although characteristics of the lesion may have an impact on the decision regarding the approach. The anterior surgical approach has been well established and is a good open approach for patients with large benign masses or cysts. Trans vesical, per vesical or retro vesical approach could be considered [1]. In other reports of cystadenoma PSA were normal [2, 4] but in our case PSA was 11 that the diagnosis was very difficult because the huge mass was at the prostatic anatomic and prostatic cancer was in differential diagnosis. But after the surgery PSA was normal and has no rising again. In this respect our case was absolutely unique. The tumor was resected using an open trans vesical approach that enabled full exposure of the seminal vesicle without damaging the nerves and blood supply of the bladder that also routinely done by other surgeon [5, 6]. Due to the rarity of these tumors, no standard surgical approach can be concluded but in literature this surgery was done with laparoscopy and robot assisted laparoscopy [2, 3].

## Conclusion

Surgical intervention may be considered when a cystadenoma of the seminal vesicle is diagnosed and symptoms or tumor growth occurs transvesical removal of seminal vesicle mass is an effective surgical procedure for seminal vesicle disease.

## Competing interests

The authors declare no competing interest.
